# Serum BDNF and progression to MCI in cognitively normal older adults A prospective cohort study

**DOI:** 10.1016/j.tjpad.2025.100254

**Published:** 2025-06-20

**Authors:** Gary A. Rosenberg

**Affiliations:** Center for Memory and Aging, University of New Mexico Health Sciences Center, Albuquerque, NM 87131, United States

The rapid advances in instruments to measure proteins in the blood has revealed a number of novel biomarkers in patients with Alzheimer’s disease (AD). The majority of the biomarkers are detrimental. In this issue of the journal, Kim and colleagues [1] report a potentially protective protein. Using a large Korean cohort of elderly individuals that have been studied at entrance into the study and 4 years later, they show that

Brain Derived Neurotrophic factor (BDNF) is elevated in the subjects that did not progress from normal cognition to mild cognitive impairment (MCI). They attribute this to a possible protective role of BDNF. Their findings are similar to those of others showing that BDNF reduces the progression to AD. This data is unique because of the long follow-up time.

Brain-derived neurotrophic factor (BDNF) plays an important role in neuronal survival and growth, serves as a neurotransmitter modulator, and participates in neuronal plasticity, which is essential for learning and memory. Thus, the data in this paper is consistent with earlier laboratory findings in animals. This is a descriptive study that sheds little light on the underlying mechanism of the protective effect.

Females, less well educated, and younger age were the ones showing the effect. While the authors attempt to explain these observations, they will need to be studied further in larger groups. The number of subjects were small to start with and then they were divided into even smaller groups, making the statistical analysis questionable. This means that the study could be underpowered and larger studies will be needed to establish the validity of the findings.

## CRediT authorship contribution statement

**Gary A. Rosenberg:** Writing – review & editing.

## Declaration of competing interest

The authors declare that they have no known competing financial interests or personal relationships that could have appeared to influence the work reported in this paper.
